# Effects of an interprofessional Quality Circle-Deprescribing Module (QC-DeMo) in Swiss nursing homes: a randomised controlled trial

**DOI:** 10.1186/s12877-021-02220-y

**Published:** 2021-05-01

**Authors:** Damien Cateau, Pierluigi Ballabeni, Anne Niquille

**Affiliations:** 1 Community Pharmacy, Center for Primary Care and Public Health (Unisanté), University of Lausanne, Rue du Bugnon 44, CH-1011 Lausanne, Switzerland; 2 School of Pharmaceutical Sciences, University of Geneva, Geneva, Switzerland; 3 Institute of Pharmaceutical Sciences of Western Switzerland, University of Geneva, University of Lausanne, Geneva, Switzerland

**Keywords:** Deprescribing, Nursing home, Quality circle, Collaboration, Potentially inappropriate medications

## Abstract

**Background:**

Potentially inappropriate medications (PIMs) are common among nursing homes (NH) residents, as is polypharmacy. Deprescribing has emerged in the past decade as a safe and effective way to reduce the use of PIMs and improve patient outcomes. However, effective deprescribing interventions are expensive, as they require specialised staff and a great amount of time for each resident.

The Quality Circle Deprescribing Module (QC-DeMo) intervention was designed to be less resource-intensive than medication reviews, the current deprescribing gold standard. It consists of a QC session in which physicians, nurses, and pharmacists define a local deprescribing consensus for specific PIMs classes, which is then implemented in the NH. The intervention was trialled in a RCT, with the NH as unit of analysis.

**Methods:**

After randomisation, intervention NHs enacted the QC-DeMo at the start of the follow-up year. The primary outcomes were the proportion of PIM galenic units and number of PIM defined daily dose per average resident and per day (DDD/res). PIM status was assessed by a combination of the 2015 Beers list and the Norwegian General Practice-Nursing Home criteria. Secondary outcomes were the number of DDD/res to avoid and to reevaluate; safety outcomes were mortality, hospitalisations, falls, and use of physical restraints. Outcomes were evaluated at follow-up using linear regression models, adjusting for the outcome baseline values.

**Results:**

Fifty-eight NHs took part in the trial; no individual residents were recruited. The intervention did not reduce the primary outcomes, but a strong trend towards reduction was seen for the number of PIM DDD/res, which accounts for the doses used. PIM DDD/res to reevaluate were significantly reduced, mostly through a reduction in the use of proton-pump inhibitors. Falls and use of physical restraints were not affected, but a statistical interaction between the mission of the NH (geriatric unit or specialised dementia unit) and the intervention group was seen for mortality and hospitalisations.

**Conclusions:**

The QC-DeMo intervention can reduce the use of some PIM classes, and could usefully complement other deprescribing interventions.

**Trial registration:**

ClinicalTrials.gov (NCT03688542), registered on 26.09.2018, retrospectively registered.

**Supplementary Information:**

The online version contains supplementary material available at 10.1186/s12877-021-02220-y.

## Background

Potentially inappropriate medications (PIMs), i.e. drugs likely to cause more harm than benefits, and polypharmacy (the use of five or more drugs) are both highly prevalent in the geriatric population, and especially so in older people living in nursing homes (NHs) [1–4]. These prescribing practices have been independently associated with negative health outcomes such as falls, adverse drug events, hospitalisations, and death [5]. Taking action to reduce both polypharmacy and the use of PIMs is thus warranted.

Deprescribing interventions have been proven to be effective and safe at doing so: recent meta-analyses showed that, both in the community and in NHs, deprescribing reduces polypharmacy and the use of PIMs, and has a positive impact on falls and mortality [6, 7]. In both meta-analysis, the most effective interventions were patient-centred medication reviews, a time-consuming intervention requiring specialised clinicians, which may not be available in every setting.

In the NHs of the Swiss canton of Vaud, an integrated pharmacist service (IPS) has been active since 2010; in the neighbouring canton of Fribourg, a similar IPS was active between 2002 and 2018. This IPS consists, for the most part, of regular meetings (quality circles, QC) between the pharmacists, physicians and nurses active in a NH, with the goal of producing local prescribing consensus to improve drug use [8]. This approach led, in particular, to a reduction in drug costs and improved antibiotics stewardship [9, 10].

This collaborative environment seemed well-suited to test a novel deprescribing intervention, less resource-intensive than medication reviews: a Quality Circle Deprescribing Module (QC-DeMo). We hypothesised that this approach could lead to a reduction in the use of PIMs, without increasing mortality, hospitalisations, falls, or the use of restraints, and without requiring scarce resources, such as clinical pharmacists, to perform medication reviews. As such complex interventions are often difficult to transfer into routine practice, the QC-DeMo trial also evaluated the implementation process.

## Methods

### Research project

The QC-DeMo trial was part of the Opportunities and Limits to Deprescribing in Nursing Homes (OLD-NH) research project, aiming 1) to quantify the use of PIMs in Swiss NHs with an active IPS and 2) to understand residents’, relatives’ and professionals’ view of deprescribing, in order to 3) design and trial deprescribing interventions to reduce the use of PIMs. Results for the first two parts of the project have been published [11–13].

The complete protocol for the QC-DeMo trial, including a nested trial of an Individual Deprescribing Intervention (IDeI), has been published [14]. Results from the implementation evaluation will be published separately.

### Population

All NHs from the cantons of Fribourg and Vaud caring for a geriatric population and with an IPS active for at least 1 year at the time of recruitment were eligible for participation. All involved professionals (physician, head nurse, pharmacist) and the NH direction had to give written agreement to take part in the study.

Recruitment of NHs was performed by the investigators through direct contact with their pharmacists, who then discussed participations with the other professionals involved. In case of insufficient participation, a second recruitment round was planned 1 year later.

### Randomisation and blinding

Participating NHs were clustered according to their physicians (some physicians attend multiple NHs) to prevent contamination; clusters were then randomised by between the intervention and control groups (ratio 1:1), using a random list generated by the investigators with the Stata statistical package (v15, Stata Corp, College Station LLC, TX, USA). The investigators informed NHs of allocation by e-mail after randomisation.

Given the nature of the intervention, neither the investigators, NHs, nor healthcare professionals were blinded; only the statistician performing the analysis was blinded.

### Intervention

The intervention consists of a QC session focused on ways to deprescribe specific drug classes, resulting in the creation of a local deprescribing consensus. Before the start of the trial, pharmacists of the NHs allocated to the intervention group participated in a half-day education session covering the evidences supporting deprescribing specific drug classes (e.g. proton-pump inhibitors urinary spasmolytics, etc.), and presenting clinical tools to facilitate it. The investigators provided presentation templates to participating pharmacists, in order to enhance fidelity to the intervention. Pharmacists selected the classes to cover in the QC session based on drug use in the NH, and then organised and facilitated the session, and formalised the resulting deprescribing consensus, possibly addressing multiple drug classes. Participants in the QC session were encouraged to devise implementation strategies for the consensus, including who should be responsible for each step.

**Fig. 1:**
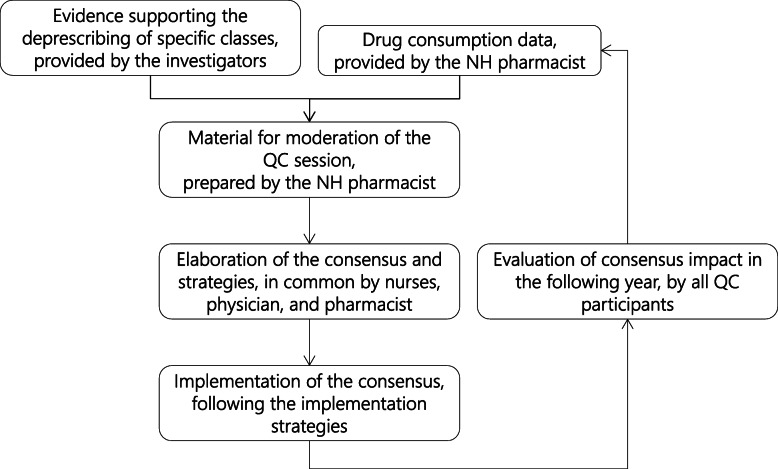
Process of the QC-DeMo intervention; QC-DeMo: Quality Circle Deprescribing Module; NH: nursing home; QC: quality circle

The QC-DeMo session took place between December 2017 and January 2018 for round 1 and December 2018 and January 2019 for round 2, with the consensus being enacted in February 2018, respectively February 2019, at the latest. Participating NHs could hold supplementary sessions during the year if necessary to review or update the consensus. The consensus and strategies were reviewed by all QC session participants during the annual feedback session on drug consumption for the previous year, held in all NHs each year in March. Figure 1 details the intervention process.

The investigators collected and compiled the consensus from all NHs and, after anonymisation, forwarded them to all NHs in the intervention group to foster discussion and ideas around deprescribing.

### Comparator

NHs allocated to the control group cared for their residents as usual and maintained the regular IPS activities. No restrictions were made to the topics that could be discussed during the regular QC sessions held in the NHs of the control group. They were offered the possibility to enact the intervention after study completion, but this was not mandated.

### Outcomes

The primary outcome is the proportion of galenic units (tablet, ml of solution, gram of cream) considered potentially inappropriate used at follow-up. Appropriateness was assessed according to the combination of the Beers’ list (2015 edition) [15] and of the Norwegian General Practice – Nursing Home criteria (NORGEP-NH) [16]. The Beers list was chosen for its widespread use in research and clinical practice and NORGEP-NH because it is, to the best knowledge of the authors, the only European list specifically designed for use with NH residents. Following NORGEP-NH’s classification, drugs present in either list, as single drug or in combination, were classified as *Avoid* (“Regular use should be avoided” category in NORGEP-NH, unconditional “Avoid” recommendation in the Beers’ list) or *Reevaluate* (“Deprescribing criteria” category in NORGEP-NH, recommendation to avoid in specific circumstances in the Beers’ list). The list of drugs considered potentially inappropriate can be found in Additional file 1. Due to the limits of the available data, some criteria in NORGEP-NH and the Beers’ list could not be implemented, as discussed in detail in [11].

During the course of the trial, and before any analysis had been carried out, work on another aspect of the OLD-NH project enabled the use of a more robust and more sensitive outcome: the number of potentially inappropriate defined daily dose (DDD) per average resident and per day (DDD/res). The methodology for computing this outcome has been published [11]. The number of potentially inappropriate DDD/res has thus been added as co-primary outcome; the protocol has been amended accordingly.

Secondary outcomes are the number of DDD/res to avoid and to reevaluate. Safety outcomes are the number of hospital days and falls per average resident and per year, the mortality rate, and the rate of use of physical restraints (e.g. bed straps, vests, chair bars).

### Data collection

Data on drug consumption and the number of days spent in each NH were provided by the central monitoring team for the IPS; data on hospitalization, falls, use of restraints, and death were collected directly by the investigators in the NHs.

All data were annualized and aggregated at the NH level; no data on individual residents were collected.

### Sample size

Data obtained by investigators on the use of PIMs in the NHs of the canton of Fribourg showed that 22.8% (standard deviation (SD) 6.3%) of galenic units could be considered potentially inappropriate in 2015. Based on data of other deprescribing trials [17–19], we hypothesised a 20% relative reduction in the proportion of potentially inappropriate galenic units in the intervention group, or 4.6% in absolute term. We estimated the year-on-year Pearson correlation coefficient at 0.3, and hypothesised that the SD of the reduction in PIM use would be equal between groups. Hence, 66 NHs would need to be included to discriminate between a mean differences of 0 in the control group and 4.6% in the intervention group (with risks of α and β errors of 5 and 20%).

### Statistical analysis

Upon checking for model assumptions, all outcomes were assessed with linear regression models, estimating the effect of group at follow-up under adjustment for the baseline outcome value, NH average number of residents at baseline, and NH mission (geriatric unit or specialised dementia unit). The cantons of Fribourg and Vaud had, at the time of study, a different model of drug provision (ambulatory in Vaud, and similar to a hospital model in Fribourg); particularly, pharmacists had no economic incentive to provide more drugs in the NHs of Fribourg, whereas they did in Vaud. We hypothesised that this difference could impact the effect of the intervention, and all models were thus also adjusted for the canton of the NH.

Some NHs participating in the trial care for older adults with dementia-related issues (psycho-geriatric NHs), which could impact the ability of NH staff to implement the consensus resulting from the intervention. Therefore, for all analyses, the existence of an interaction between the mission and the randomisation group was checked for, and included in the final model if significant; mission and group were included only as main effects if not. Clustering by GPs was not taken into account for the analysis, as only nine clusters contained more than one NH. All pre-specified analysis were performed according to intention to treat; the 5% significance level was considered.

Exploratory analysis were performed to assess the effect of the intervention on specific drug classes, using the same methodology as for the co-primary outcome and the same statistical method. Not all intervention NHs formalised a consensus for every drug class; therefore, for these exploratory analysis, only the NHs in the intervention group that formalised a consensus on the considered class were included in the analysis, and compared to all NHs from the control group, akin to a per-protocol analysis.

### Ethical considerations and reporting

The QC-DeMo trial was submitted to the Cantonal (Vaud) Ethics Committee; as no data on individual residents were collected, ethical approval for this trial was waived (decision 2017–01009). The trial was registered on ClinicalTrials.gov (NCT03688542), where the protocol is available.

The CONSORT extensions for the reporting of pragmatic trials and the reporting of non-pharmacologic treatments trials were followed for the preparation of this article [20, 21].

## Results

### Characteristics of the NHs

**Fig. 2:**
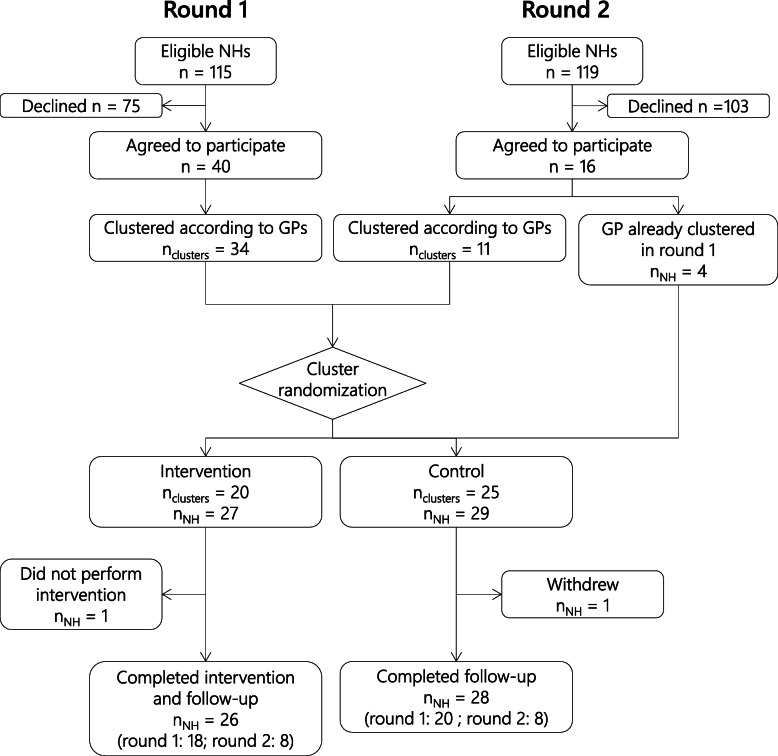
Flow-chart for the QC-DeMo trial. GP: general practitioner; NH: nursing home. Round 1 joined in 2017, round 2 in 2018

Fifty-six NHs agreed to take part in the QC-DeMo trial, 40 in round 1 and 16 in round 2 (Fig. 2). Recruitment for round 1 was carried out between August and October 2017, and between July and October 2018 for round 2. All NHs that declined to participate cited a lack of time, either of the nursing team, their physician, or their pharmacist, as the main reason. Participating NHs were representative of their population in terms of size (average number of beds in these two cantons is 52) and distribution between cantons and missions (42, respectively 140, NHs in Fribourg and Vaud in 2018) [22]; their characteristics are resumed in Table 1.

**Table 1:** Baseline characteristics of included NHs

	Intervention ***n*** = 27	Control ***n*** = 28
Canton (n, % of all included NHs)		
Vaud	21 (38%)	23 (41%)
Fribourg	6 (11%)	6 (11%)
NH mission (n, % of all included NHs)		
Geriatric	16 (29%)	19 (35%)
Psycho-geriatric ^a^	11 (20%)	9 (16%)
Number of average residents	48 [33–81]	35 [22–47]
Number of GPs	2 [1–3]	1 [1–2]
Proportion of potentially inappropriate galenic units	26% [20–30%]	22% [6–45%]
Number of potentially inappropriate DDD ^b^	2.4 [1.8–2.7]	2.4 [1.9–2.7]
Of which to avoid ^b^	0.3 [0.2–0.5]	0.4 [0.2–0.5]
Of which to reevaluate ^b^	1.9 [1.6–2.3]	2.1 [1.6–2.3]
Mortality rate	32% [26–42%]	34% [26–48%]
Days in hospital ^c^	2.7 [1.5–3.4]	2.5 [1.5–3.8]
Number of falls ^c^	2.3 [1.7–3.3]	2.3 [1.4–3.1]
Rate of physical restraint use	23% [12–39%]	27% [12–45%]

*NH* nursing home; all data are median [IQR], unless otherwise specified; ^a^: including NHs caring for both geriatric and psycho-geriatric residents; ^b^: reported per average resident and per day. ^c^: reported per average resident and per year

Twenty NH clusters were randomised to the intervention group, and 25 to the control group; 6 NHs from the second round were not randomised, but directly assigned to an existing cluster from round 1, as they shared at least one GP with this cluster. This resulted in 27 NHs allocated to the intervention group, and 29 to the control group (see Fig. 2). Randomisation resulted in an imbalance between groups regarding the average number of residents and the distribution of missions; these variables were adjusted for in all statistical analysis. One NH in the intervention group did not perform the intervention due to a severe health issue of its pharmacist, and one withdrew from the control group before initial data collection; the former was included in all analysis, and the latter excluded, as no data were collected.

All NHs having held the deprescribing QC session defined local consensus for one or more therapeutic class (see Table 2); the most common were addressing the use of proton-pump inhibitors (21/26 NHs), statins (17/26) and benzodiazepines (10/26). An example of consensus and accompanying strategies can be found in Additional file 2.

**Table 2:** Drug classes addressed in formalised consensus

Therapeutic class (ATC code)	Rationale for deprescribing ^**a**^	Number of NH (%) ***n*** = 26 ^**b**^
Proton-pump inhibitors (A02BC)	Frequent overprescribing; side-effects in case of long-term use	21 (78%)
Lipid modifying agents (C10)	Negative risk/benefit ratio in people aged 85 or more if used in primary prevention	17 (63%)
Benzodiazepines (N05B & N05C)	Side effects in case of long-term use	10 (37%)
Urinary spasmolytics (G04BD) and anticholinergic drugs	Lack of efficacy (urinary spasmolytics); frequent side effects (all anticholinergics)	9 (33%)
Glucose-lowering drugs (A10B)	Higher HbA1C targets for very old patients; risk of adverse events if blood sugar too low	9 (33%)
Antihypertensives (C03, C07, C08, C09)	Higher blood pressure targets for very old patients	8 (30%)
Bisphosphonates (M05BA & M05BB)	Lack of evidence for efficacy after 5+ years of treatment	6 (22%)
Anti-dementia drugs (N06D)	Lack of efficacy; high costs	6 (22%)
Antidepressants (N06A)	Frequent overprescribing	6 (22%)
Antipsychotics (N05A)	Lack of evidence for use in behavioural and psychological symptoms of dementia	5 (19%)

*ATC* Anatomic Therapeutic Chemical classification, *NH* nursing home, *HbA*_*1C*_ glycated haemoglobin; n (% of column); ^a^: as presented in the education session; ^b^: 1 NH did not perform the intervention

At baseline, 26% of galenic units used in the NHs of the intervention group were considered potentially inappropriate, and 22% in the control group. The average resident of these NHs received a median of 2.4 potentially inappropriate DDD in both groups, with no difference between the NHs of the two recruitment rounds. Most of these potentially inappropriate DDD/res were drugs to reevaluate (median 1.9 and 2.1 in the intervention and control groups), with only 0.3, respectively 0.4, DDD/res to avoid.

### Impact of the intervention

The effect of the intervention on the proportion of galenic units considered potentially inappropriate (primary outcome) was inconclusive (*p* = 0.240 for a difference between groups at follow-up); data were compatible with a reduction of the number of potentially inappropriate DDD per resident (co-primary outcome) in the intervention group, however a degree of uncertainty about our estimation remains (*p* = 0.083, see Table 3). Detailed results for all models are available in Additional file 3.

**Table 3:** Effect of the intervention on the outcomes

	Regression coefficient	95% confidence interval	*p*-value
Primary outcomes			
% of PIM galenic units	−0.014	[−0.038;+0.010]	0.240
Number of PIM DDD/res	−0.183	[−0.392;+0.025]	0.083
Secondary outcomes			
Number of DDD/res to avoid	−0.035	[−0.095;+0.025]	0.252
Number of DDD/res to reevaluate	−0.237	[− 0.435;−0.040]	0.020
Safety outcomes			
Mortality rate ^a^			
Intervention group	−12.7%	[−21.5%;−4.0%]	0.005
Geriatric mission	+8.7%	[+0.8%;+16.6%]	0.032
Group × mission	−11.4%	[−23.3%;+0.05%]	0.060
Days in hospital (*n* = 51) ^a,b^			
Intervention group	+1.551	[+0.164;+2.938]	0.029
Geriatric mission	−1.381	[−2.670;−0.091]	0.036
Group × mission	+1.910	[+ 0.020;+3.799]	0.048
Number of falls (*n* = 46) ^b^	−0.165	[−0.754;+0.424]	0.575
Rate of physical restraints use (*n* = 46)	−4.2%	[−16.0%;+7.6%]	0.479

All data are difference between intervention and control group at follow-up, estimated using linear regression models, under adjustment for outcome baseline value, canton, mission and size of the NH. *n* = 55, unless otherwise specified. *PIM* potentially inappropriate medication, *DDD/res* defined daily dose per average resident and per day. ^a^: also adjusted for the interaction between group and mission, as it proved significant. ^b^: reported per average resident and per year

The number of potentially inappropriate DDD/res to avoid was not statistically significantly changed by the intervention, whereas a statistically significant change was seen in the number of DDD/res to reevaluate. This group comprises mostly of long-term use drugs, like cardiovascular medication, and drugs in which overuse is frequent, like proton-pump inhibitors or neuroleptics. From a baseline of 1.9 DDD/res considered to reevaluate, the intervention resulted in a diminution of 0.24 DDD/res in the intervention group compared to the control group, a 13% reduction.

Neither the number of falls nor the rate of use of restraint were significantly impacted by the intervention (Table 3). For hospitalisations, a statistically significant interaction between group and mission was found and included in the final model. For mortality, the interaction neared statistical significance (*p* = 0.06), and was also included in the final model. Regression models predicted, for NHs of the intervention group with a psycho-geriatric mission, a lower mortality rate (26.9%, CI_95_ [20.9%; 32.9%]) and a higher number of days spent in hospital per average resident and per year (3.8, CI95 [2.8; 4.7]), compared to all other NHs (Table 4).

**Table 4:** Predicted values for mortality rates and hospitalisations

	Mortality rate (p for interaction = 0.060)		Mean hospitalisation days per average residents and per year (p for interaction = 0.048)	
	Predicted value	95% confidence interval	Predicted value	95% confidence interval
Intervention group, Geriatric mission	35.6%	[30.4%;40.8%]	2.4	[1.6;3.3]
Intervention group, Psycho-geriatric mission	26.8%	[20.1%;32.9%]	3.8	[2.8; 4.7]
Control group, Geriatric mission	36.9%	[31.9%;41.9%]	2.8	[2.0;3.5]
Control group, Psycho-geriatric mission	39.6%	[33.2%;46.0%]	2.2	[1.2;3.3]

Values predicted at follow-up by the regression model under adjustment for baseline value, canton, mission, size of NH, randomisation group, and group × mission interaction

For all outcomes, no statistically significant difference was seen between the two cantons, except for the number of hospital days per average resident and per year, which was higher in Vaud than in Fribourg (+ 2.0, CI_95_ [+ 0.9; + 3.2], *p* = 0.001).

In exploratory analyses, the effect of the intervention on specific drug classes were assessed in the NHs that held a session and formalised a consensus for this class, compared to the whole control group. The intervention had a statistically significant effect on the use of proton-pump inhibitors (ATC A02BC): from a baseline value of 0.32 DDD/res, use was reduced by 0.07 DDD/res in the intervention group (CI_95_ [− 0.123;-0.010], *p* = 0.022 for difference with control group); no significant effect of the intervention was found for the other classes. Detailed results can be found in Additional file 3.

## Discussion

Putting into practice the consensus devised during the deprescribing-focused quality circle session did not significantly reduce the proportion of potentially inappropriate galenic units, the primary outcome. However, as described in the amended protocol [14], this outcome cannot reflect some of the changes that could result from the intervention, such as lowering the doses of PIMs used. For this reason, a more robust outcome, the number of potentially inappropriate DDD per resident, was included as co-primary outcome during the trial. While a definitive conclusion on the effects of the intervention on this parameter cannot be reached at the moment, as the statistical significance threshold was not reached, data are compatible with a reduction of PIMs use in the intervention group, driven by a significant decrease in the use of PIMs of the “reevaluate” category.

This category comprises long-term use drugs, which are inappropriate in some situation either because long-term use can cause significant harm (e.g. proton pump inhibitors (PPIs), nitrofurantoin), or because the rationale for long-term use is lacking in geriatric patients (e.g. antihypertensives, statins), particularly in primary prevention in patients with limited life expectancy. Most deprescribing guidelines and recommendations are focused on such drugs [23, 24], and the intervention could be a useful way to implement deprescribing for these classes. The success in reducing the use of PPIs signals that this approach could be successful for class-specific deprescribing.

However, for the other drug classes for which an exploratory analysis was performed, the intervention did not produce a significant change in use (see Additional file 4). A possible reasons for this is that the intervention would not work on these specific drugs; this seems doubtful, as the intervention was successful in reducing the use of PPIs, which are notoriously hard to reduce [25], as they provide symptomatic relief, whereas statins, the second-most widely discussed class during the QC session, have none. Another possible explanation is that the drug classes most frequently discussed during the QC were already very sparsely used; the low number of DDD/res at baseline for two classes, statins and urinary spasmolytics, supports this hypothesis: statins, with 0.14 DDD/res, were used at an average therapeutic dose by a small minority of residents, and by an even smaller number if high doses were prescribed, as is recommended in secondary prevention [26]. Even fewer urinary spasmolytics were used at baseline. Inversely, benzodiazepines, the third most-discussed class (0.31 DDD/res at baseline), were used in the same amount than PPIs, but their use did not significantly change following the intervention; this could be due to the difficulty of stopping such drugs, given their well-known addictive effect. Both facts could indicate that the intervention was targeted towards the wrong drug classes, and could be more effective if focused on more drugs that widely-used and have low addictive effects, like antihypertensives or antidepressants [11]. In this trial, few NHs chose to work on these drug classes. A hypothesis is that pharmacists chose drug classes to discuss in the QC session based more on the perceived feasibility of deprescribing, and less on the relevance of these classes in their NHs.

A final possibility for the lack of effect of the intervention on the classes other than PPIs is that the consensus produced following the QC session were not put into practice. This hypothesis will be explored in the implementation study that was carried on in parallel to the present clinical trial, and will be reported in a forthcoming publication.

We hypothesised that the intervention would result in a 20% reduction, in relative term, in the use of PIMs. This hypothesis was based on the result of previous deprescribing trials, in particular the one conducted by Potter and colleagues, in which 19.7% of regular drugs used by NH residents were successfully discontinued [17]. QC-DeMo produced a reduction in the use of PPIs of the same magnitude, but only a 13% decrease in the use of PIMs to reevaluate. This is coherent with the findings of Kua and colleague’s and Page and colleague’s meta-analysis, that showed that deprescribing interventions were most effective when targeting individual residents, and not institutions [6, 7]. The fact that an effect on the use of some PIMs was seen during the QC-DeMo trial indicates that these two approaches, QC sessions and medication reviews, could be complementary: the QC-DeMo trial could be used to curb the use of specific PIMs classes in a cost-effective way, and medication reviews could be deployed to improve the care of specific residents.

The effects of QC-DeMo on mortality and hospitalizations are conflicting: significantly reducing mortality in the subgroup of NHs caring for patients with age-related psychiatric problems and, at the same time, increasing hospitalizations in these same NHs, is coherent neither with the small effect of the intervention on PIMs, nor with what is known of the effect of other deprescribing interventions in this population [7]. In particular, as, in Switzerland, NH residents that die during a temporary hospitalization are tallied with deaths occurring in the NH, any detrimental effect of the intervention would be expected to be reflected both in hospitalizations and deaths, not just on one or the other. As these conflicting effects were, moreover, only seen in one subgroup of NHs, we advise to interpret results for these two outcomes with great caution, and chose to interpret them as indicating the intervention producing neither benefit nor harm. This interpretation is corroborated by the fact that neither beneficial nor harmful effects were seen for the other safety outcomes, falls and use of physical restraints.

We hypothesised that the differences in the drug provision financing model between the two cantons could play a role in the willingness of some actors to deprescribe, as pharmacists have a financial incentive to provide more drugs in the canton of Vaud. However, this factor did not prove to be statistically significant for outcome related to drug use, indicating that the model of drug provision does not influence the effects of the intervention (see Additional file 3). The effect of the canton was seen only in the model concerning hospitalisations, indicating a possible difference in practice on this point, possibly resulting from different standards for transfering a resident to the hospital in the two cantons.

### Strength and limitations

This trial had numerous strengths: it made use of an existing, well-established collaborative practice between clinicians that enabled the implementation of a novel deprescribing intervention with only minimal training for the pharmacists. To the author’s best knowledge, it is the first trial to explore the impact of a QC-type intervention to address PIMs use; other studies, such as the COME-ON project in Belgium, incorporated interdisciplinary meetings as part of complex interventions, but did not report the effects of the separate components of the intervention [27].

The use of a co-primary outcome taking into account the dose of drug used allowed us to capture effects of the intervention that other, more widely-used outcomes, such as prevalence of PIMs or the proportion of residents successfully discontinuing one drug, could not.

This trial also suffers from many limitations. First, it did not reach its recruitment target, resulting in a lack of statistical power, possibly reflected in the absence of a statistically significant difference between the two groups for the primary outcomes.

Second, the intervention was designed to let pharmacists chose the drug classes to discuss in the QC session and on which to devise a deprescribing consensus; this was intended to enable adaptation to the local conditions and take into account possible previous deprescribing efforts in the NHs. However, it resulted in dispersion of the intervention, with most NHs choosing to work on relatively low-use drugs classes, like statins or urinary spasmolytics, instead of more widely-used ones.

By grouping the baseline years for the two rounds, our analysis also assumes that no significant change in PIMs use occurred between 2017 and 2018, but a previous study using the same methodology for outcome computation showed that such a change does occur year over year [11]. This change is, however, small (− 0.03 DDD/res per year) compared to both baseline values (Table 1) and change at follow-up for the intervention group (Table 3), and should not have significantly affected the results. The randomisation, while conducted according to the protocol, resulted in an imbalance between groups regarding the mission and size of the NHs; this was accounted for in the analysis by adjusting for these parameters. Nonetheless, the interaction found between NH mission and their allocation group in the analysis of mortality and hospitalisation indicates that, for some parameters, the intervention’s effects vary between missions, possibly because of differences in residents’ characteristics. For future studies, these differences should be taken into account by collecting more details on the NHs’ population to enable more meaningful comparison; a randomisation scheme stratified by mission should also be employed.

Two potential confounding factors were not addressed in our analyses: first, the prior efforts of the NHs to deprescribe PIMs or limit polypharmacy could have constrained the effect of the intervention. These efforts were collected as part of the implementation study, but not in a way that enabled their inclusion in the statistical models. Second, the qualifications of their attending physicians could have modified the intervention’s effect, as NHs attended by geriatricians may have been more proactive in limiting PIMs use prior to the study than those attended by a general practitioner, and more receptive to the pharmacists’ messages.

Finally, a major limitation of this trial is its lack of resident-level outcomes. While not a perfect outcome, the prevalence of PIMs is widely reported and part of relevant core outcome sets [28, 29]; being able to report it would have facilitated the comparison of our intervention with others found in the literature. More importantly, the lack of access to resident-level data prevented us from identifying problems not severe enough to require hospitalisation, but still harmful to the residents, like gastric bleeding following the deprescribing of PPIs. Specific questions to physicians, nurses or pharmacists of participating NHs about harms to the residents resulting from the application of the consensus could have usefully completed the evaluation of this intervention.

## Conclusion

The QC-DeMo intervention proved effective at reducing the use of some PIMs, notably PPIs, but did not affect the use of other inappropriate drugs, and did not reduce overall use. It could usefully complement interventions like medication review, which have proven positive effects on patient-important outcomes like mortality, as a way to reduce long-term risks of PIM use, particularly those in relation to overuse. Further studies of QC-DeMo should be conducted to better evaluate its impact on widely-used drugs to reevaluate, like antihypertensives.

## Supplementary Information

**Additional file 1.** List of ATC codes considered PIMs.

**Additional file 2.** Example of consensus and implementation strategy.

**Additional file 3.** Detailed results of regression models.

**Additional file 4.** Exploratory analysis of the impact of the intervention on specific drug classes.

## Data Availability

The datasets generated during and analysed during this trial, as well as the code used to analyse it, are available from the corresponding author upon reasonable request.

## Abbreviations

ATC: Anatomical Therapeutic Chemical classification
CER-VD: Commission cantonale (VD) d’éthique de la recherche sur l’être humain
CI_95_: 95% Confidence Interval
DDD/res: Defined Daily Dose per average resident and per day
DDD: Defined Daily Dose
IDeI: Individual Deprescribing Intervention
IPS: Integrated Pharmacist Service
NH: Nursing Home
NORGEP-NH: Norwegian General Practice – Nursing Home criteria
OLD-NH: Opportunities and Limits to Deprescribing in Nursing Homes
PIM: Potentially Inappropriate Medication
PPI: Proton Pump Inhibitors
QC: Quality Circle
QC-DeMo: Quality Circle Deprescribing Module
SD: Stadard Deviation
