# Supplementary material for: Effects of an interprofessional Quality Circle-Deprescribing Module (QC-DeMo) in Swiss nursing homes: a randomised controlled trial
Source: BMC Geriatr. 2021 May 1;21:289. doi: 10.1186/s12877-021-02220-y (PMC8088558; doi:10.1186/s12877-021-02220-y)
Supplement: Supplementary file 2 — Additional file 2. Example of consensus and implementation strategy. [file 12877_2021_2220_MOESM2_ESM.pptx]

## Slide 1
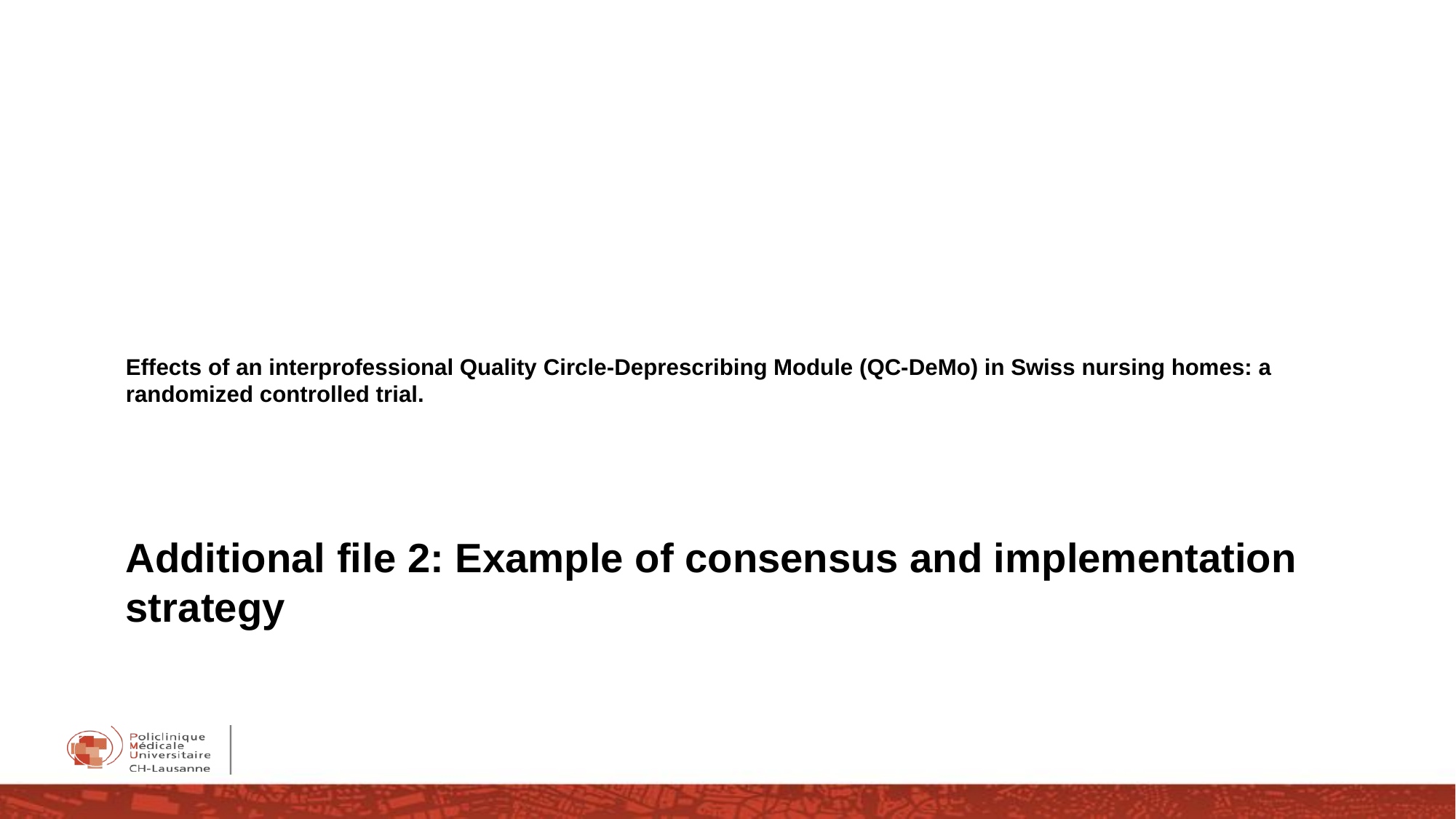

Effects of an interprofessional Quality Circle-Deprescribing Module (QC-DeMo) in Swiss nursing homes: a randomized controlled trial.
# Additional file 2: Example of consensus and implementation strategy

## Slide 2
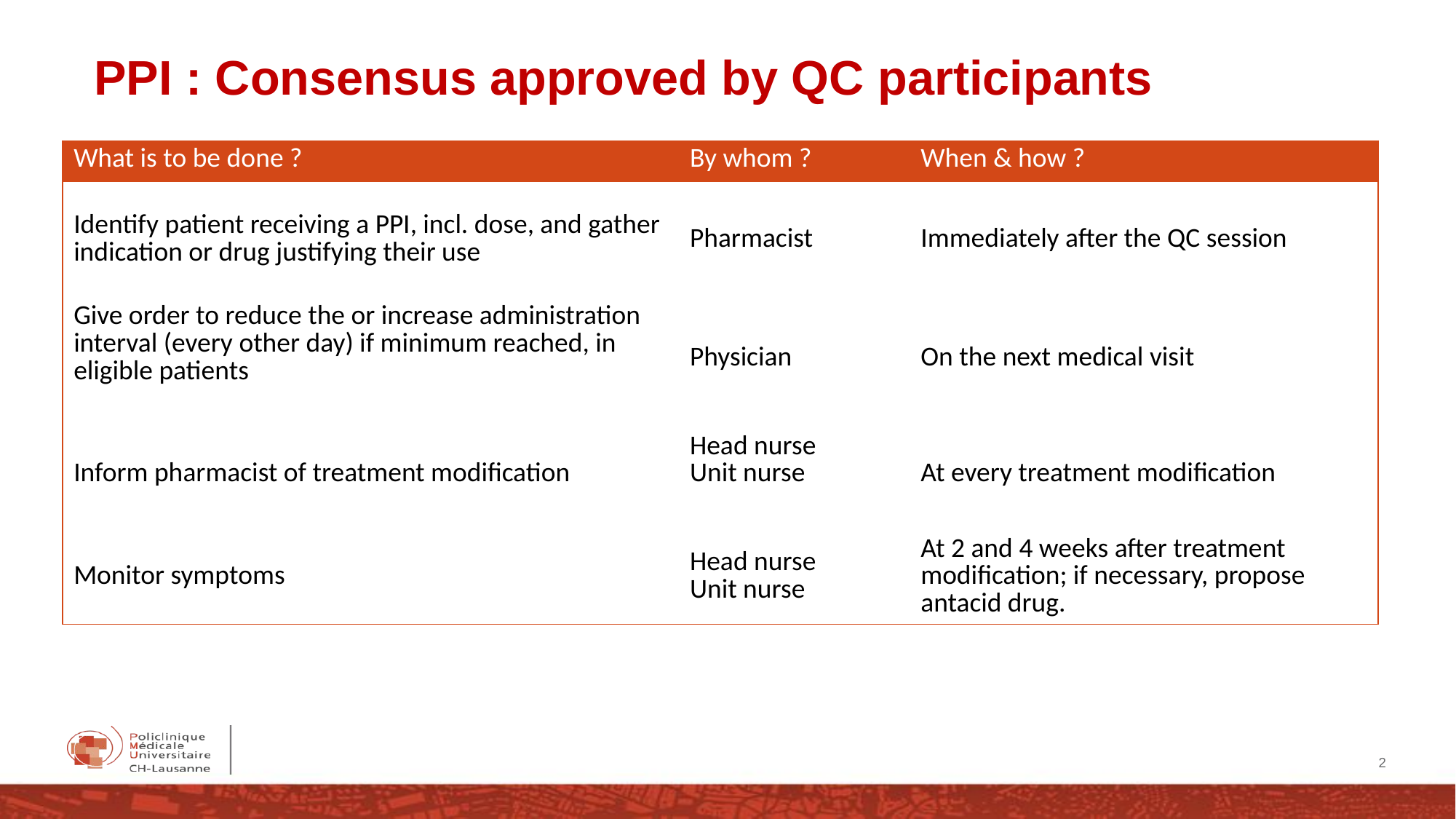

# PPI : Consensus approved by QC participants
| What is to be done ? | By whom ? | When & how ? |
| --- | --- | --- |
| Identify patient receiving a PPI, incl. dose, and gather indication or drug justifying their use | Pharmacist | Immediately after the QC session |
| Give order to reduce the or increase administration interval (every other day) if minimum reached, in eligible patients | Physician | On the next medical visit |
| Inform pharmacist of treatment modification | Head nurse Unit nurse | At every treatment modification |
| Monitor symptoms | Head nurse Unit nurse | At 2 and 4 weeks after treatment modification; if necessary, propose antacid drug. |
2

## Slide 3
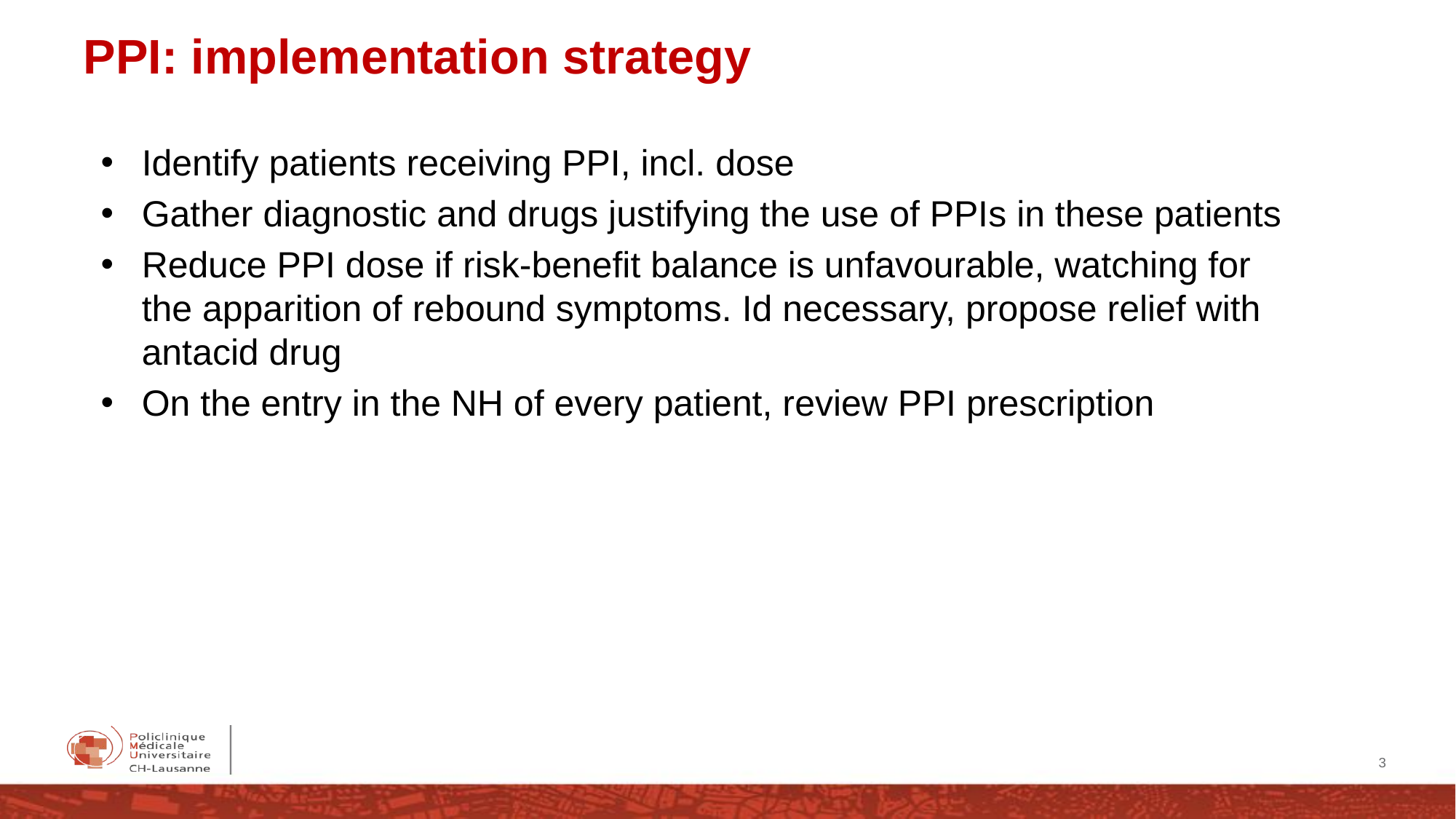

# PPI: implementation strategy
Identify patients receiving PPI, incl. dose
Gather diagnostic and drugs justifying the use of PPIs in these patients
Reduce PPI dose if risk-benefit balance is unfavourable, watching for the apparition of rebound symptoms. Id necessary, propose relief with antacid drug
On the entry in the NH of every patient, review PPI prescription
3
