# Supplementary material for: Effects of an interprofessional Quality Circle-Deprescribing Module (QC-DeMo) in Swiss nursing homes: a randomised controlled trial
Source: BMC Geriatr. 2021 May 1;21:289. doi: 10.1186/s12877-021-02220-y (PMC8088558; doi:10.1186/s12877-021-02220-y)
Supplement: Supplementary file 3 — Additional file 3. Detailed results of regression models. [file 12877_2021_2220_MOESM3_ESM.docx]

# Additional file 3: Detailed results of regression models

## Primary outcome: proportion of potentially inappropriate galenic units

|  | Regression coefficient | 95% confidence interval | p-value |
| --- | --- | --- | --- |
| Group |  |  |  |
| Control^a^ | - | - | - |
| Intervention | -0.014 | -0.038; +0.010 | 0.240 |
| Baseline value | +0.832 | +0.686; +0.979 | 0.000 |
| Canton |  |  |  |
| FR^a^ | - | - | - |
| VD | +0.010 | -0.017; +0.037 | 0.458 |
| Mission |  |  |  |
| Psycho-geriatric^a^ | - | - | - |
| Geriatric | -0.014 | -0.037; +0.009 | 0.231 |
| Average number of residents | +0.000 | -0.000; +0.000 | 0.720 |
| Constant | +0.024 | -0.047; +0.096 | 0.492 |

a: reference category

## Co-primary outcome: number of potentially inappropriate Defined Daily Dose per average resident and per day

|  | Regression coefficient | 95% confidence interval | p-value |
| --- | --- | --- | --- |
| Group |  |  |  |
| Control^a^ | - | - | - |
| Intervention | -0.183 | -0.391; +0.025 | 0.083 |
| Baseline value | +0.689 | +0.504; +0.873 | 0.000 |
| Canton |  |  |  |
| FR^a^ | - | - | - |
| VD | +0.074 | -0.177; +0.324 | 0.557 |
| Mission |  |  |  |
| Psycho-geriatric^a^ | - | - | - |
| Geriatric | +0.084 | -0.1213; +0.324 | 0.420 |
| Average number of residents | +0.003 | -0.001; +0.008 | 0.109 |
| Constant | +0.213 | -0.373; +0.008 | 0.469 |

a: reference category

## Secondary outcome 1: number of potentially inappropriate Defined Daily Dose to **avoid** per average resident and per day

|  | Regression coefficient | 95% confidence interval | p-value |
| --- | --- | --- | --- |
| Group |  |  |  |
| Control^a^ | - | - | - |
| Intervention | -0.034 | -0.095; +0.025 | 0.252 |
| Baseline value | +0.773 | +0.590; +0.954 | 0.000 |
| Canton |  |  |  |
| FR^a^ | - | - | - |
| VD | +0.026 | -0.565; +0.110 | 0.525 |
| Mission |  |  |  |
| Psycho-geriatric^a^ | - | - | - |
| Geriatric | -0.009 | -0.068; +0.050 | 0.761 |
| Average number of residents | +0.000 | -0.001; +0.001 | 0.852 |
| Constant | +0.004 | -0.166; +0.174 | 0.961 |

a: reference category

## Secondary outcome 2: number of potentially inappropriate Defined Daily Dose to **reevaluate** per average resident and per day

|  | Regression coefficient | 95% confidence interval | p-value |
| --- | --- | --- | --- |
| Group |  |  |  |
| Control^a^ | - | - | - |
| Intervention | -0.237 | -0.435; -0.040 | 0.020 |
| Baseline value | +0.624 | +0.427; +0.823 | 0.000 |
| Canton |  |  |  |
| FR^a^ | - | - | - |
| VD | +0.089 | -0.135; +0.314 | 0.428 |
| Mission |  |  |  |
| Psycho-geriatric^a^ | - | - | - |
| Geriatric | +0.047 | -0.148; +0.242 | 0.628 |
| Average number of residents | +0.005 | +0.001; +0.009 | 0.016 |
| Constant | +0.202 | -0.283; +0.688 | 0.407 |

a: reference category

## Safety outcome 1: mortality rate

|  | Regression coefficient | 95% confidence interval | p-value |
| --- | --- | --- | --- |
| Group |  |  |  |
| Control^a^ | - | - | - |
| Intervention | -12.7% | -21.5%; -4.0% | 0.005 |
| Baseline value | +40.0% | +23.7%; +56.3% | <0.001 |
| Canton |  |  |  |
| FR^a^ | - | - | - |
| VD | +4.11% | -3.2%; +5.2% | 0.264 |
| Mission |  |  |  |
| Psycho-geriatric^a^ | - | - | - |
| Geriatric | +8.7% | +0.8%; +16.7% | 0.032 |
| Mission × Group |  |  |  |
| Intervention × Geriatric | - |  |  |
| Intervention × Psycho-geriatric | - |  |  |
| Control × Geriatric | - |  |  |
| Control × Psycho-geriatric | -11.4% | -23.3%; +0.1% | 0.060 |
| Average number of residents | -0.0% | -0.2%; +0.0% | 0.278 |
| Constant | +12.1% | +0.0%; +24.2% | 0.049 |

a: reference category;

## Safety outcome 2: number of hospital day per average resident and per year

|  | Regression coefficient | 95% confidence interval | p-value |
| --- | --- | --- | --- |
| Group |  |  |  |
| Control^a^ | - | - | - |
| Intervention | +1.551 | +0.165; +2.938 | 0.029 |
| Baseline value | +0.166 | -0.150; +0.481 | 0.295 |
| Canton |  |  |  |
| FR^a^ | - | - | - |
| VD | +2.037 | +0.866; +3.208 | 0.001 |
| Mission |  |  |  |
| Psycho-geriatric^a^ | - | - | - |
| Geriatric | -1.381 | -2.670; -0.091 | 0.036 |
| Mission × Group |  |  |  |
| Intervention × Geriatric | - |  |  |
| Intervention × Psycho-geriatric | - |  |  |
| Control × Geriatric | - |  |  |
| Control × Psycho-geriatric | +1.910 | +0.020; +3.780 | 0.048 |
| Average number of residents | +0.001 | -0.019; +0.021 | 0.885 |
| Constant | +1.567 | -0.156; +3.290 | 0.074 |

a: reference category;

## Safety outcome 3: number of falls pare average resident and per year

|  | Regression coefficient | 95% confidence interval | p-value |
| --- | --- | --- | --- |
| Group |  |  |  |
| Control^a^ | - | - | - |
| Intervention | +0.286 | -0.522; +1.095 | 0.479 |
| Baseline value | +0.755 | +0.444; +1.067 | 0.000 |
| Canton |  |  |  |
| FR^a^ | - | - | - |
| VD | +0.156 | -0.899; +1.212 | 0.766 |
| Mission |  |  |  |
| Psycho-geriatric^a^ | - | - | - |
| Geriatric | -0.364 | -1.148; +0.418 | 0.353 |
| Average number of residents | -0.003 | -0.019; +0.013 | 0.678 |
| Constant | +1.272 | -0.413; +2.958 | 0.135 |

a: reference category

## Safety outcome 4: rate of physical restraints use

|  | Regression coefficient | 95% confidence interval | p-value |
| --- | --- | --- | --- |
| Group |  |  |  |
| Control^a^ | - | - | - |
| Intervention | -0.042 | -0.160; +0.076 | 0.479 |
| Baseline value | +0.660 | +0.465; +0.854 | 0.000 |
| Canton |  |  |  |
| FR^a^ | - | - | - |
| VD | -0.001 | -0.160; +0.158 | 0.988 |
| Mission |  |  |  |
| Psycho-geriatric^a^ | - | - | - |
| Geriatric | -0.026 | -0.137; +0.085 | 0.639 |
| Average number of residents | +0.002 | -0.000; +0.004 | 0.094 |
| Constant | +0.047 | -0.187; +0.282 | 0.685 |

a: reference category
