# Supplementary material for: Effects of an interprofessional Quality Circle-Deprescribing Module (QC-DeMo) in Swiss nursing homes: a randomised controlled trial
Source: BMC Geriatr. 2021 May 1;21:289. doi: 10.1186/s12877-021-02220-y (PMC8088558; doi:10.1186/s12877-021-02220-y)
Supplement: Supplementary file 4 — Additional file 4. Exploratory analysis of the impact of the intervention on specific drug classes. [file 12877_2021_2220_MOESM4_ESM.docx]

# Additional file 4: Exploratory analysis of the impact of the intervention on specific drug classes

| Specific therapeutic classes (ATC code) | Baseline value for intervention group, DDD/res (mean, SD) | Difference vs control group at follow-up | 95% confidence interval | p-value |
| --- | --- | --- | --- | --- |
| Proton-pump inhibitors (A02BC, n=21) | 0.32 (0.12) | -0.067 | [-0.123;-0.010] | 0.022 |
| Lipid modifying agents (C10, n=17) | 0.14 (0.07) | -0.017 | [-0.069;+0.035] | 0.508 |
| Benzodiazepines  (N05B & N05C, n=10) | 0.31 (0.21) | -0.020 | [-0.080;+0.040] | 0.502 |
| Urinary spasmolytics (G04BD, n=9) | 0.04 (0.04) | -0.010 | [-0.036;+0.015] | 0.411 |
| Glucose-lowering drugs (A10B, n=9) | 0.10 (0.03) | -0.018 | [-0.057;+0.021] | 0.354 |
| Antihypertensives  (C03, C07, C08, C09, n=8) | 1.18 (0.52) | -0.104 | [-0.277;+0.070] | 0.233 |
| Bisphosphonates (M05BA & M05BB, n=6) | 0.02 (0.02) | +0.001 | [-0.010;+0.013] | 0.817 |
| Anti-dementia drugs (N06D, n=6) | 0.10 (0.05) | +0.014 | [-0.032;+0.061] | 0.537 |
| Antidepressants  (N06A, n=6) | 0.41 (0.09) | -0.056 | [-0.191;+0.080] | 0.410 |
| Antipsychotics  (N05A, n=5) | 0.21 (0.19) | +0.003 | [-0.047;+0.052] | 0.915 |

Analysis carried out only in NHs having formalized a consensus for this class, compared to all 28 NHs in the control group. n is the number of NHs that formalized a consensus on this class. ATC: Anatomic Therapeutic Chemical classification; DDD: Defined Daily Dose; DDD/res: DDD per average resident and per day.
